# Poly(Aspartic Acid) Functionalized Poly(ϵ-Caprolactone) Microspheres with Enhanced Hydroxyapatite Affinity as Bone Targeting Antibiotic Carriers

**DOI:** 10.3390/pharmaceutics12090885

**Published:** 2020-09-17

**Authors:** Stijn G. Rotman, Thomas F. Moriarty, Benjamin Nottelet, Dirk W. Grijpma, David Eglin, Olivier Guillaume

**Affiliations:** 1 AO Research Institute Davos, 7270 Davos Platz, Switzerland; stijn.rotman@aofoundation.org (S.G.R.); fintan.moriarty@aofoundation.org (T.F.M.); david.eglin@aofoundation.org (D.E.); 2 Department of Biomaterials Science and Technology, Faculty of Science and Technology and Technical Medical Centre, University of Twente, 7522 NB Enschede, The Netherlands; d.w.grijpma@utwente.nl; 3 IBMM, University of Montpellier, CNRS, ENSCM, 34093 Montpellier, France; benjamin.nottelet@umontpellier.fr; 4 Institute of Materials Science and Technology, TU Wien, 1060 Vienna, Austria

**Keywords:** drug delivery, poly(aspartic acid), bone infection, bone targeting, bone seeking agents

## Abstract

Bone infection is a feared complication for patients with surgically fixed bone fractures and local antibiotic delivery is important in prophylaxis and treatment of these infections. Recent studies indicated that *Staphylococcus aureus* can penetrate bone tissue through micron-sized canaliculi and evade systemic and currently available local antibiotic treatments. Targeting bacteria within the bone requires highly efficient delivery of antimicrobials to the infected bone tissue. In this work, a biodegradable microsphere carrier loaded with antibiotics and with specific affinity to bone mineral was developed. Two widely used antibiotics, i.e., Gentamicin-dioctyl sulfosuccinate (GM-AOT) and Ciprofloxacin (CF) were embedded in poly(ϵ-caprolactone) (PCL) microspheres fabricated by oil-in-water emulsion techniques with carboxylated poly(vinyl alcohol) (cPVA) as surfactant. The carboxylic acid groups present at the Poly(ϵ-caprolactone)/cPVA (PCL-cPVA) microsphere surface were functionalized with aspartic acid oligomers (ASP) granting bone targeting properties. We report on cPVA synthesis, microsphere formulation, and antibiotic loading of PCL/cPVA-ASP microspheres. Antibiotic loaded PCL/cPVA-ASP microspheres show sustained release of its antibiotic load and can inhibit bacterial growth in vitro for up to 6 days. PCL/cPVA-ASP microspheres show enhanced affinity to mineralized substrates compared to non-functionalized PCL/cPVA microspheres. These findings support further development of these bone targeting antibiotic carriers for potential treatment of persistent bone infections.

## 1. Introduction

In orthopedic surgery, osteomyelitis (OM) is a dreaded complication that can affect up to 27% of patients with bone fractures [1]. It is generally accepted that a rigorous debridement of infected bone combined with local and systemic antibiotics is necessary to achieve the highest chance of successful treatment outcome. Nonetheless, for approximately 30% of patients that undergo such extensive therapies, infection re-emerges within 12 months [2]. Gram-positive *Staphylococcus aureus* (*S. aureus*) [3] and Gram-negative *Escherichia coli* (*E. coli*) [4] are two main pathogens involved in adult OM. It has been recently shown that *S. aureus* has the ability to deform and penetrate (sub-)micron structures in vitro [5] and this was also observed microscopically in clinical bone biopsies [6]. This phenomenon is expected to contribute to the high recurrence rate of OM as bacteria residing in bone canaliculi may evade local and systemic antibiotic therapies without causing tissue necrosis and so may easily be missed during surgical debridement procedures.

The presence of bacteria deep within the bone necessitates more efficient antimicrobial delivery strategies. One approach is the direct functionalization of the antibiotic molecules with bone targeting groups, forming a bone targeting prodrug of the active antibiotic [7,8]. Despite straightforward synthesis strategies, there are some downsides to the prodrug approach [9]. The prodrug must show appropriate and controlled conversion into its therapeutically active parent drug and from a translational perspective, the prodrug would need to undergo renewed approval by regulatory institutions before clinical trials can be considered.

An alternative strategy for enhanced local antibiotic delivery is to load antibiotics into carrier constructs that can release the drug payload in the vicinity of the infection site over a suitable period [10]. For several decades, antibiotic loaded bulk materials like poly(methylmethacrylate) (PMMA) and collagen have been used as carrier materials in orthopedic surgery and have improved post-operative outcomes significantly [11]. Due to the non-biodegradable nature of PMMA, a second surgical procedure is needed to retrieve these materials after antibiotic release. Alternatively, collagen carriers (e.g., GENTA-COLL^®^ and Collatamp^®^G) are biodegradable, but most of these materials are characterized by their very rapid release of antibiotic load [12]. Some other biodegradable bulk constructs can offer a more sustained release of antibiotic loads, for example poly(trimethylene carbonate) materials, and remove the need for an extraction surgery [13,14]. However, a common feature with all these bulk materials is the inability to localize themselves consistently at the bone interface. Antimicrobials loaded within micro or nanoparticles might offer an advantage in this regard [15].

Several attempts have been made to design micro- and nanoparticles for sustained antibiotic delivery [16,17], in which delivery systems were endowed with bone affinity through incorporation of bisphosphonate groups [18]. There are several technologies available for solid microsphere fabrication [19,20], which include evaporation-based methods such as electrospray [21] or O/W emulsions and microfluidic methods or based on polymerization of polymeric emulsions [22] for the formation of crosslinked solid microspheres. In this work, a mechanically established O/W emulsion followed by solvent evaporation was selected as a scalable and straightforward approach for the fabrication of large quantities of microspheres.

We previously reported on an antibiotic delivery system consisting of Alendronate (ALN) functionalized poly(ϵ-caprolactone) (PCL) microspheres in which hydrophobic Gentamicin-dioctyl sulfosuccinate (GM-AOT) was embedded in the PCL polymer matrix [18]. However, ALN is known to inhibit osteoclast formation and bone mineral resorption [23] with several ALN-related clinical side effects reported such as bisphosphonate-related osteonecrosis of the jaw (BRONJ) [24], and so alternative bone targeting groups are preferred. Just like ALN, aspartic acid (ASP) oligomers can act as a chelator and interact with divalent ions like Ca^2+^ that are present in inorganic bone matrix, but do not display any secondary effects such as those associated with bisphosphonates. Studies regarding the chelation efficacy of ASP oligomers have reported that oligomers of six or more ASP monomers show the highest affinity to bone minerals [25]. During an investigation of estradiol-ASP prodrugs, residence time of ASP oligomers at the bone site was shown be prolonged by incorporating d-configurated ASP monomers into the oligomers, because d-ASP is not hydrolysable under physiological conditions in contrast to l-ASP [25]. ASP oligomers have been used to endow bone affinity to radionuclides [26] and poly(lactic-co-glycolic acid) (PLGA) particles [27]. Implementation of ASP oligomers for bone targeted antibiotic therapy has, to the best of our knowledge, not been reported yet. In this work we aimed to develop an oil/water (O/W) emulsion-based production of antibiotic loaded PCL microspheres with (d-ASP)_6_ oligomers grafted on the surface via carboxylated PVA (cPVA). Once produced, we further characterize microsphere size and antibiotic loading efficiency as well as their affinity to hydroxyapatite (HAP). Assessment of elution of two antibiotics from the microspheres and their ability to kill Gram-positive and Gram-negative planktonic bacteria and prevent bacterial growth were also studied. Finally, the advantages of ASP functionalized microspheres as antibiotic delivery systems to bone were examined in terms of establishing high local concentrations at a bone mimicking hydroxyapatite surface.

## 2. Materials and Methods

### 2.1. Materials

Poly(vinyl alcohol) (PVA; M_W_ = 30,000 g·mol^−1^; 100% hydrolyzed,), poly(ϵ-caprolactone) (PCL; M_W_ = 80,000 g·mol^−1^), 2-(N-morpholino)ethanesulfonic acid (MES), 1-Ethyl-3-(3-dimethylaminopropyl)carbodiimide (EDC), *N*-hydroxysuccinimide (NHS), phosphate-buffered saline (PBS), succinic anhydride, dioctyl sulfosuccinate sodium salt (AOT), and ciprofloxacin (CF) were purchased from Sigma Aldrich, Steinheim, Germany. Gentamicin sulphate, 4-Dimethylaminopyridine (4-DMAP), ethanol, 2-propanol, acetone, and dichloromethane (DCM) were purchased from Roth (Karlsuhe, Germany). d-(Aspartic acid)_6_ oligomers (ASP) were synthesized on demand by GenScript (Leiden, The Netherlands). Tryptin Soy Agar (TSA) plates were purchased from Oxoid AG (Basel, Switzerland). CellTiter Blue^®^ cell viability assay was purchased from Promega, Madison, WI, USA. Bone marrow-derived mesenchymal stem cells (BMSC) were isolated from human bone marrow donated by the Inselspital Bern. Osteoassay plates were acquired from Corning (Amsterdam, The Netherlands). The bacterial strains used were methicillin susceptible *S. aureus* JAR 890 (CCOS, Wädenswill, Switzerland) and *E. coli* O76:H51 (CCOS, Wädenswill, Switzerland).

### 2.2. Hydrophobic Gentamicin (GM-AOT) Synthesis

GM-AOT was synthesized as previously reported by ter Boo et al. [14] In short, equal volumes of 0.40% *w/v* gentamicin sulphate in buffer (10 mM sodium acetate, KCl and CaCl_2_ at pH = 5) and 1.25% *w/v* dioctyl sodium sulfosuccinate (AOT) in DCM were mixed by vigorous stirring for 3 h. The two phases dissociated for 30 min and GM-AOT was isolated from the DCM by evaporation of the solvent. Further characterization including Fourier transform infrared spectroscopy (FTIR) and the water solubility of GM-AOT was previously described [18].

### 2.3. Carboxylated PVA Synthesis

The cPVA synthesis procedure was adapted from Zhang et al. [28]. Briefly, PVA (5 g) was added to 50 mL ddH_2_O and heated to 90 °C under magnetic stirring using an oil bath to allow for complete solubilization of PVA. The temperature of the PVA solution was cooled to 65 °C and 1.39 g 4-DMAP was added under moderate stirring. After 1 h, 11.36 g of succinic anhydride was added to the PVA solution and stirred at 65 °C for 24 h. The reaction was cooled to room temperature and the polymers were precipitated in 500 mL acetone and washed a second time in 200 mL acetone. The polymers were dried overnight under reduced pressure at a temperature of 40 °C. The carboxylated PVA (cPVA) product was analyzed by nuclear magnetic resonance (^1^H-NMR, Bruker Avance AV 500) in D_2_O solvent with a field strength of 500 MHz to assess the degree of carboxylation.

### 2.4. Carboxylated PVA Cytotoxicity

PVA and cPVA were dissolved ddH_2_O. Minimum Essential Medium (MEM) was prepared by dissolving the MEM powder in the PVA and cPVA solutions and diluting the polymer concentration with MEM. Finally, 10% Fetal Bovine Serum (FBS) and 1% penicillin/streptomycin (p/s) was added to finalize the PVA supplemented mediums. Human bone marrow derived mesenchymal stem cells (BMSC’s) were cultured in absence of PVA or cPVA in a 48-well plate with a seeding density of 5000 cells/well until a confluency of 70–80% was reached. Then, the mediums were aspirated and 400 µL of PVA supplemented medium was added to the cells (*n* = 6). After 24 h the PVA supplemented mediums were aspirated and a CellTiter Blue^®^ cell viability assay was performed according to manufacturer’s instructions.

### 2.5. PCL/cPVA Microsphere Fabrication

A total of 500 mg PCL was dissolved in 5 mL DCM. When drug was incorporated, 125 mg of GM-AOT, CF, or pyrene was added to the PCL solution. For formulations containing CF, 1 mL of acetic acid was added as a miscible solvent in order to solubilize the antibiotic. Then, 100 mL of 1% aqueous cPVA solution that acted as a surfactant, was pH adjusted to 8.0 with 1M NaOH to ensure a favorable environment for the carboxylic acid groups of cPVA to be in a deprotonated state, in which they act as better surfactants. The polymer solution was added dropwise, under a vortex, to 10 mL of the cPVA solution over a period of approximately 1 min. This pre-emulsion was sonicated using a sonication probe (Bandelin Sonopuls GM70, Berlin, Germany) for three bursts of 30 s and finally added to the remaining 90 mL of cPVA solution. The DCM could evaporate under ambient conditions with mild magnetic stirring for 4 h. The solidified microspheres were filtered with a cell-strainer (mesh size of 70 µm) in order to remove agglomerates and washed twice in double distilled water (ddH_2_O) to remove cPVA that remained in solution. The PCL/cPVA microspheres were resuspended in 10 mL ddH_2_O, flash frozen in liquid nitrogen, and lyophilized.

### 2.6. PCL/cPVA Microsphere Surface Functionalization with ASP

**Figure 1:**
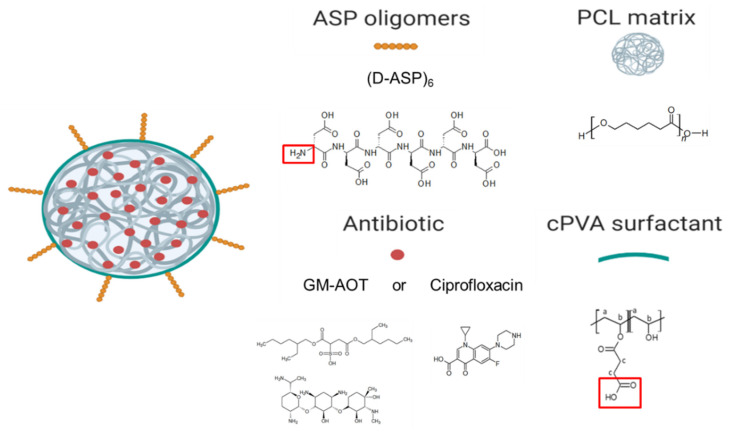
Schematic of PCL/cPVA-ASP microspheres loaded with antibiotic. PCL was used as a biodegradable material for the matrix of the microspheres. The antibiotic CF and the ion-counter ion complex GM-AOT were embedded in the PCL microspheres. ASP oligomers are conjugated to the microsphere cPVA surfactant through EDC/NHS conjugation of the carboxylic acids of cPVA and the primary amine group of the *N*-terminus of ASP (both boxed in red).

An EDC/NHS solution was made by dissolving 100 mg EDC and 100 mg NHS in 10 mL MES-buffer at a pH of 5.5. The lyophilized PCL/cPVA microspheres (100 mg) were fully resuspended in 10 mL EDC/NHS solution by probe sonication. EDC/NHS activation of the carboxylic acid groups on the microspheres was allowed for 45 min at 4 °C. The microspheres were centrifugated and washed with ddH_2_O to remove excess EDC/NHS. Next, the microspheres were dispersed in a 10 mg/mL ASP oligomer aqueous solution at neutral pH and the dispersion was stirred mildly for 2 h at room temperature. Finally, the functionalized microspheres were collected by centrifugation and washed with ddH_2_O. The PCL/cPVA-ASP microspheres were flash frozen in liquid nitrogen and lyophilized. A schematic of the final microsphere product is presented in Figure 1.

### 2.7. Scanning Electron Microscopy (SEM) of PCL/cPVA-ASP Microspheres

Freeze-dried PCL-cPVA-ASP microspheres loaded with GM-AOT or CF were dispersed in ethanol and 40 µL was deposited on scanning electron microscopy (SEM) specimen mounts covered with an adhesive tape. After evaporation of the ethanol, a 10 nm layer of Au/Pd was deposited on the samples by sputter-coating. SEM imaging was performed immediately after on a field emission scanning electron microscope (S-4700, Hitachi, Zürich, Switzerland) with a beam voltage of 1.0 keV. At least 100 microspheres’ diameters were measured from the SEM images using standard Axiovision4 software (version 4.9.1.0). The size polydispersity index (PDI) of the microspheres was calculated by Equation (1).

(1) $$\mathrm{PDI}={\left(\mathrm{Average}\;\mathrm{diameter}/\mathrm{Standard}\;\mathrm{deviation}\right)}^{2}$$

### 2.8. Antibiotic Encapsulation Efficiency and Antibiotic Release from PCL/cPVA-ASP Microspheres

PCL/cPVA-ASP microspheres loaded with GM-AOT or CF were disintegrated by dispersing the microspheres in 0.1M NaOH solution for several hours. Solutions containing the GM-AOT load were subsequently analyzed by complexation with o-phthaldialdehyde reagent, followed by adsorption measurement (λ = 332 nm) using a spectrophotometer (MultiskanGo, Thermo Scientific, Waltham, MA, USA) [29]. CF encapsulation was determined by high pressure liquid chromatography (HPLC) analysis using a mobile phase of 2% Acetic acid/Acetonitrile (84:16) under a flow rate of 1 mL/min and a detection wavelength of 280 nm. To assess the drug quantity embedded inside the microspheres, the embedding efficacy (EE%) can be determined following Equation (2). The drug load (DL%) of the microspheres can be calculated with Equation (3).

(2) $$\mathrm{EE}\%=W_{\mathrm{DM}}/W_{\mathrm{DA}}\times 100\%$$

(3) $$\mathrm{DL}\%=W_{\mathrm{DM}}/W_{T}\times 100\%$$

With W_DM_ as the weight of the drug embedded in the microsphere as determined by HPLC or colorimetric o-phthaldialdehyde assay, W_DA_ as the weight of the drug added to the O/W emulsion during particle fabrication and W_T_ as the total weight of the drug loaded microsphere sample. All EE% and DL% values were determined by averages of technical triplicates (*n* = 3).

To establish antibiotic release profiles, accurately weighed quantities (10 mg) of microspheres were dispersed in 1 mL PBS by probe sonication (*n* = 3). To allow for the collection of the complete 1 mL of supernatant, the dispersions were centrifuged with a desktop centrifuge (ROTILABO, Roth, Karlsruhe, Germany) at 9000× *g* after 1, 3, and 5 h and 1, 2, 3, 4, 7, 8, 9, and 13 days of incubation at 37 °C on an orbital shaker. The supernatant was replaced with 1 mL fresh PBS, followed by redispersion of the microspheres by sonication. Care was taken not to disturb the microsphere pellet during supernatant sampling. GM-AOT and CF concentrations in the collected supernatants were determined as described above.

### 2.9. Antimicrobial Effects on Planktonic Bacteria

The opacity of bacterial dispersions upon addition of GM-AOT or CF loaded PCL/cPVA-ASP was monitored over 48 h in order to assess the antimicrobial effect of released GM-AOT or CF. Bacterial suspensions of *S. aureus* and *E. coli* was initially set at an optical density at 600 nm (OD_600_) of 0.5 in PBS. In triplicate, 5 mg of GM-AOT loaded microspheres were added to 1 mL of *S. aureus* dispersion and 5 mg of CF loaded microspheres were added to 1 mL of *E. coli* dispersion (*n* = 3). The samples were gently resuspended and the OD_600_ was assessed at regular intervals to assess the reduction in OD_600_ caused by the bactericidal effect of the released antibiotic. Control groups for the OD measurements included bacterial dispersions of both bacterial strains without added microspheres and dispersions of GM-AOT and CF loaded microspheres in PBS (Figure S1). For the CFU quantification, unencapsulated antibiotics in solution (0.5 mg/mL) were used as a control group (*n* = 3).

### 2.10. Zone of Inhibition of Antibiotic Loaded PCL/cPVA-ASP Microspheres

PCL/cPVA-ASP microspheres loaded with GM-AOT or CF were dispersed in PBS at a concentration of 10 mg/mL and bacterial lawns of *S. aureus* or *E. coli* were streaked on TSA plates according to the Kirby–Bauer method. In the center of the TSA plate, 100 µL of the microsphere dispersions were carefully pipetted on a sterile blanc paper disc (Sensi-disc, BD Biosciences, Franklin Lakes, NJ, USA) in triplicate. GM-AOT loaded microspheres were tested against *S. aureus* bacterial lawns while CF loaded microspheres were tested on *E. coli* bacterial lawns. The plates were incubated overnight at 37 °C and 5% CO_2_ and the inhibition diameters were measured. The microsphere loaded discs were transferred to a freshly streaked TSA plate and incubated for another 24 h followed by zone of inhibition (ZOI) measurements. This process was repeated daily until no ZOI was observed.

### 2.11. PCL/cPVA-ASP Affinity with Hydroxyapatite Surfaces

Pyrene loaded PCL/cPVA-ASP and PCL/cPVA microspheres (5 mg/mL) were incubated in an Osteoassay 96-well plate containing thin HAP films on the bottom of the wells (*n* = 6). After 1 h, the wells were washed three times with ddH_2_O. Between each wash, the well plate was measured using a fluorescence plate reader (Viktor^3^, Perkin Elmer, Schwerzenbach, Switzerland) with a Hoechst filter (λ_ex_/λ_em_ = 353 nm/483 nm). A standard containing a known weight of Pyrene loaded PCL/cPVA-ASP microspheres was measured after every washing step to account for any leeching of the pyrene out of the microspheres. In order to visualize the microspheres on the HAP films, the Osteoassay 96-well plates were imaged using a fluorescent microscope (EVOS FL Auto 2, Thermo Fisher Scientific, Basel, Switzerland), again implementing the Hoechst filter settings.

### 2.12. Zone of Inhibition Around Hydroxyapatite Granules Incubated with PCL/cPVA-ASP

HAP granules (*n* = 4) with diameters between 5 and 10 mm were incubated for 1 h in a GM-AOT or CF loaded PCL/cPVA or PCL/cPVA-ASP microsphere dispersions at a concentration of 5 mg/mL under mild rocking conditions. The HAP granules were washed in PBS and were placed centrally on a TSA plate that was streaked with a *S. aureus* or *E. coli* bacterial lawn prepared as described above. The HAP granules were slightly pressed into the agar to ensure contact between the granule and the agar layer. After overnight incubation at 37 °C, the average zone of inhibition around the HAP granule was calculated from four measurements in different directions. Due to the varying sizes of the HAP granules, the distance from the granule surface to the inhibition front was measured, instead of the total diameter of the inhibition zone. The granules were transferred to a freshly streaked agar plate, in order to assess the zone of inhibition established by antibiotic released at day 2. This process was repeated daily until an inhibitory zone could no longer be observed.

### 2.13. Statistical Analysis

All numerical results (EE%, DL%, and microsphere diameter) are given as an average ± standard deviation (SD). Average values are presented on bar graphs and X-Y plots with the error bars representing the SD. Two sample Student’s *t*-tests were performed to indicate significance of results.

## 3. Results

### 3.1. NMR and Cytotoxicity Analysis of cPVA

**Figure 2:**
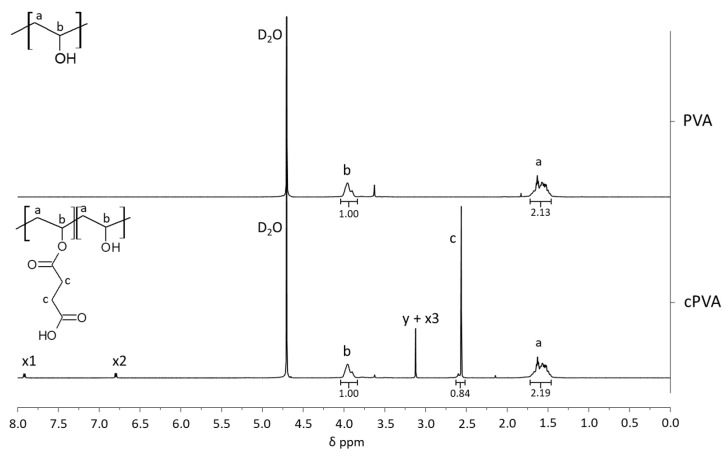
^1^H-NMR spectra of PVA and cPVA. Poly(vinyl alcohol) (PVA): ^1^H-NMR (D_2_O, 500 MHz): a = δ 1.62 (m, 2H, CH_2_), b = δ 3.96 (m, 1H, CH). Carboxylated poly(vinyl alcohol) (cPVA): ^1^H-NMR (D_2_O, 500 MHz): a = δ 1.62 (m, 2H, CH_2_), c = δ 2.56 (s, 4H, CH_2_CH_2_), b = δ 3.96 (m, 1H, CH), x = 4-Dimethylaminopyridine (4-DMAP) trace peaks, y = succinic anhydride trace peaks.

After the carboxylation process of PVA, the cPVA product was analyzed by ^1^H NMR spectroscopy of which the spectra can be seen in Figure 2. Successful grafting is presented by a peak at 2.56 ppm, caused by the protons of the methylene groups of ring-opened succinic anhydride (succinic acid) grafted on the PVA molecular backbone. The degree of substitution of PVA’s hydroxyl groups to succinic acid groups was determined to be 21 mole percent, by means of peak integration of proton signals of the PVA backbone and the methylene groups of grafted succinic acid.

**Figure 3:**
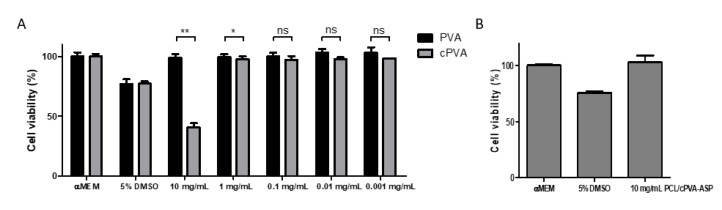
(**A**) CellTiter Blue^®^ Cytotoxicity assessment of PVA and cPVA on human bone marrow stem cells (hBMSCs) (*n* = 6). Cell viabilities for cPVA concentrations below 1 mg/mL were higher than 97% compared to α-Minimal Essential Medium (αMEM) control group. No significant differences in cell viability were observed for concentrations below 0.1 mg/mL. (**B**) CellTiter Blue^®^ Cytotoxicity assessment of hBMSCs exposed to 10 mg/mL PCL/cPVA-ASP microspheres (*n* = 6). No significant differences were observed between microspheres group and αMEM control group. Two sample Student’s t-tests were performed to indicate significant differences of results. Level of significance is indicated with the following symbols: ns = *p* > 0.01, * = *p* < 0.01 and ** = *p* < 0.001.

While PVA is considered a non-cytotoxic material, the cytotoxicity of cPVA had to be assessed as cytotoxic chemicals (e.g., 4-DMAP) were used during the carboxylation reaction and were observed in trace amounts in the NMR analysis (Figure 2). Figure 3 shows the cytotoxic effects of PVA and cPVA on hBMSCs. While the tested concentrations of PVA did not show a reduction in cell viability, cPVA did show a strong reduction at the highest concentration (10 mg/mL). This concentration is however far from the local concentrations that are reached during in vitro and in vivo experimental situations, even when large quantities of microspheres are utilized. When the hBMSCs were exposed to PCL/cPVA-ASP microspheres in a concentration of 10 mg/mL, no cytotoxic effects could be observed. It can thus be concluded that cPVA surfactants do not show cytotoxic effects in the relevant concentration range.

### 3.2. PCL/cPVA-ASP Microsphere Characteristics

**Figure 4:**
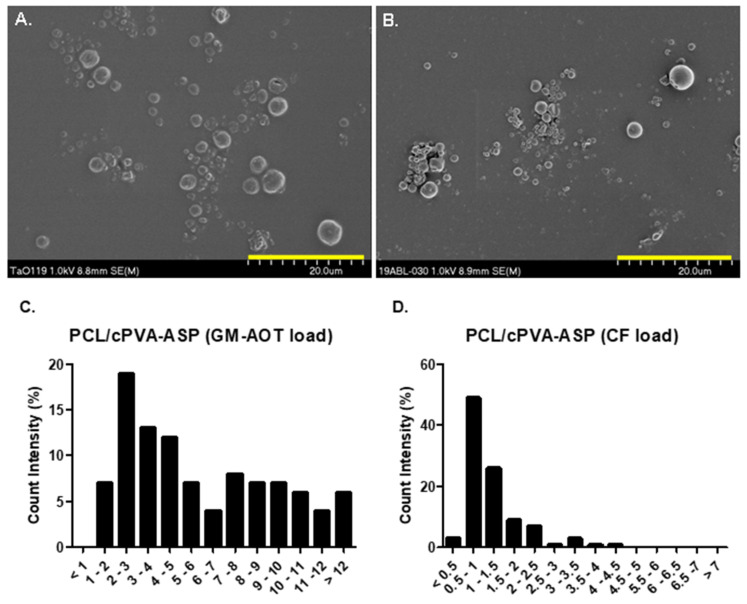
(**A**) PCL/cPVA-ASP microspheres with a GM-AOT drug load. (**B**) PCL/cPVA-ASP microspheres with a CF drug load. (**C**) Histogram showing the size distribution of PCL/cPVA-ASP microspheres with GM-AOT drug load. (**D**) Histogram showing the size distribution of PCL/cPVA-ASP microspheres with CF drug load. Yellow scalebar equals 20 µm.

After PCL/cPVA functionalization with ASP, the microspheres were imaged by SEM. Two representative images of the two groups of microspheres containing GM-AOT and CF can be seen in Figure 4A,B, respectively. Table 1 presents the average diameter of the microsphere formulations, the PDI, and the antibiotic load characteristics EE% and DL%. As the fabrication methods are identical, size averages of the microspheres were dependent on the embedded drug, with GM-AOT loaded microspheres having a diameter average of 6.03 ± 4.05 µm (*n* = 110) while CF loaded microspheres were 1.24 ± 0.76 µm (*n* = 103) in diameter. With PDI values of 0.450 and 0.373, both microsphere formulations can be considered polydisperse (PDI > 0.2). The EE% of antibiotics in PCL/cPVA-ASP microspheres were 12.76% ± 1.78% for CF and 23.77% ± 1.39% for GM-AOT. The DL% was 3.19% ± 1.53% for CF and 4.75% ± 0.28% for GM-AOT. Figure 4C,D shows the size distribution of PCL/cPVA-ASP microspheres with GM-AOT and CF drug load, respectively. A narrower size distribution was observed for CF loaded microspheres compared to the microspheres with GM-AOT load.

### 3.3. Antibiotic Release from PCL/cPVA-ASP Microspheres

**Figure 5:**
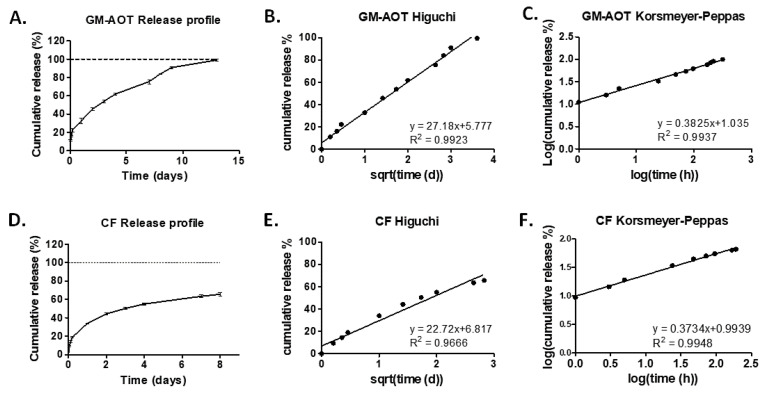
(**A**) Release profile of GM-AOT from PCL/cPVA-ASP microspheres. (**B**) GM-AOT release data represented in a Higuchi plot. (**C**) GM-AOT release data represented in a Korsmeyer–Peppas plot. (**D**) Release profile of CF from PCL/cPVA-ASP microspheres. (**E**) CF release data represented in a Higuchi plot. (**F**) CF release data represented in a Korsmeyer–Peppas plot. All data represents an average ± standard deviation of triplicate measurements.

Data on the release of CF and GM-AOT from PCL/cPVA-ASP was collected and subjected to various drug release models including the Higuchi model and the Korsmeyer–Peppas model [30]. Both models can be considered as important tools to understand which type of release kinetics take place during the elution of drug from the polymeric microspheres. Figure 4 presents the cumulative release of CF and GM-AOT (Figure 5A,D) and the same data presented in Higuchi (Figure 5B,E) and Korsmeyer–Peppas plots (Figure 5C,F). Linear trendlines were fitted to the Higuchi and Korsmeyer–Peppas datasets by means of linear regression with the fit equation and R^2^ reported for each of the graphs and are additionally presented in Table 2.

Figure 5A,D shows the cumulative release of GM-AOT and CF, respectively. The release of GM-AOT nearly reached its complete drug load after 2 weeks. CF release appeared to plateau after 1 week of antibiotic release. In Figure 5B,E, the antibiotic release data was plotted on a Higuchi axis system (square root of time vs. cumulative release), with linear fits with slopes (*K_H_*-values) of 27.18 and 22.72 and R^2^ of 0.9923 and 0.9666, respectively, presented in Table 2. This demonstrated that the drug release was predominantly diffusion controlled. The slightly poorer linear fit of the CF release data might be due to the incomplete drug release and plateauing of the release profile at approximately 60% of cumulative CF release.

Drug release of GM-AOT and CF from PCL/cPVA-ASP also fits well with the Korsmeyer–Peppas model (Figure 5C,F; r^2^ of 0.9937 and 0.9939, respectively) and the value of the slope of the curve fitting (*n*-value) yields information on how the drug load is released from the polymeric matrix (diffusion, carrier erosion, or a combination). When the *n*-value parameter modeling drug release from spherical materials is 0.43, it is considered that the drug is released in a Fickian diffusion manner. The *n*-values of GM-AOT and CF release reported in this work are 0.38 and 0.37, respectively.

### 3.4. In Vitro Antimicrobial Effects of GM-AOT and CF Loaded PCL/cPVA-ASP Microspheres

**Figure 6:**
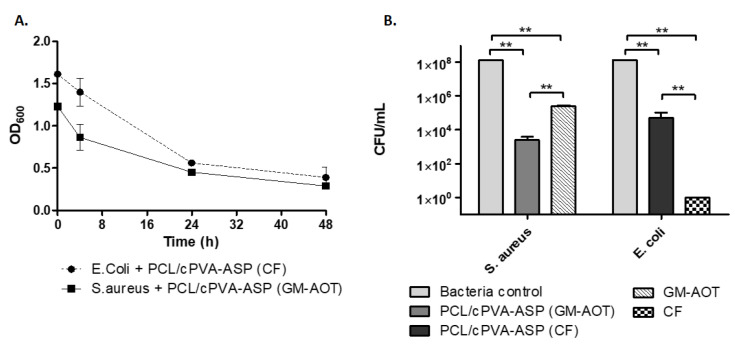
(**A**) Optical density at 600 nm of *Escherichia coli* and *Staphylococcus aureus* bacterial dispersions (*n* = 3) monitored over 48 h. (**B**) Remaining CFU concentrations of control bacterial dispersions (*n* = 3) and bacterial dispersions treated with antibiotic loaded PCL/cPVA-ASP or with free solubilized antibiotics after a 48-h incubation. Error bars indicate SD-values of triplicate values. Student’s *t*-test (α = 0.01) with the following legend: ** = *p* < 0.001.

First, antimicrobial effects were assessed by monitoring the evolution of the OD_600_ of bacterial dispersions supplemented with GM-AOT and CF loaded PCL/cPVA-ASP microspheres over time and the results are shown in Figure 6A. The OD_600_ of the relevant control groups are shown in Figure S1. The antibiotics released from the microspheres caused a sharp reduction in optical density of the dispersion. Even though bacterial dispersions at 37 °C in PBS in absence of antibiotics still causes a mild OD_600_ decrease due to a lack of bacterial nutrients or bacterial agglomeration (Figure S1), the OD_600_ decrease observed in Figure 6A is evidently much higher and thus caused by the presence of CF or GM-AOT loaded microspheres. In Figure 6B, the remaining CFU concentration of the microsphere supplemented bacterial samples and bacterial control samples are presented. A 5 log_10_ reduction of *S. aureus* and a 3.5 log_10_ reduction of *E. coli* CFU was reported, showing the strong antimicrobial effects of the antibiotics released from PCL/cPVA-ASP microspheres. Free CF could completely eradicate the *E. coli* bacterial suspension (Figure 6B), while GM-AOT was unable to do so. This most likely originates from the very low solubility of GM-AOT in the aqueous bacterial suspension. A continuous release of GM-AOT from the microspheres showed increased antimicrobial effects on *S. aureus*.

### 3.5. PCL/cPVA-ASP Affinity with Hydroxyapatite Surfaces

**Figure 7:**
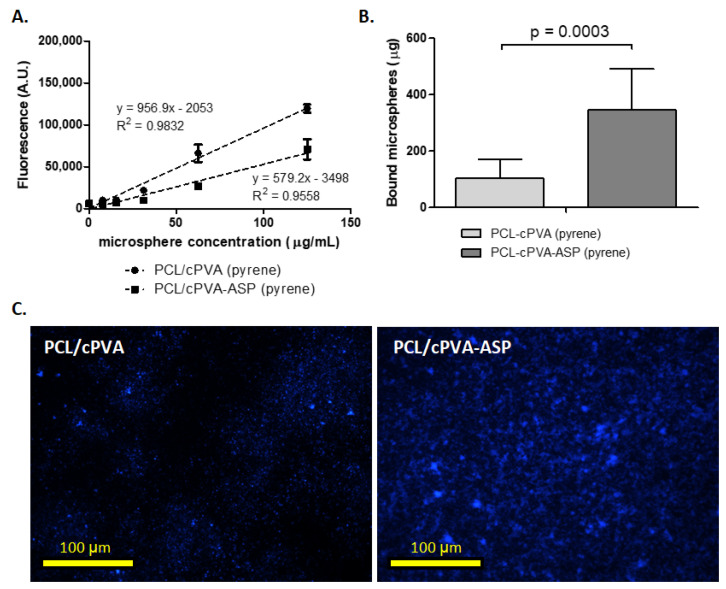
(**A**) Standard curves of the Hoechst absorption by pyrene loaded in PCL/cPVA and PCL/cPVA-ASP microspheres (*n* = 3). (**B**) Quantitative assessment of the presence of pyrene loaded microspheres on calcium phosphate surfaces established by standard curve correlation (*n* = 6). Statistical test was a paired *t*-test (α = 0.01). (**C**) Microscopic images of pyrene loaded in PCL/cPVA and PCL/cPVA-ASP microspheres on calcium phosphate surfaces.

Affinity of PCL/cPVA microspheres to HAP surfaces was increased after ASP grafting on the cPVA surfactant layer (Figure 7). To quantify microsphere presence on HAP surfaces, fluorescent pyrene was embedded in the PCL matrix. Figure 6A shows the standard curves of fluorescent signals coming from pyrene embedded in PCL/cPVA and PCL/cPVA-ASP microspheres. The reduced fluorescent signal of PCL/cPVA-ASP compared to PCL/cPVA microspheres can be explained by a loss of pyrene during the ASP functionalization procedure and highlights the importance of establishing two standard curves when assessing PCL/cPVA and PCL/cPVA-ASP fluorescent signals. Figure 7B shows the binding efficacy of PCL/cPVA and PCL/cPVA-ASP to HAP surfaces. A significant increase of PCL/cPVA-ASP microspheres present on the HAP surfaces compared to PCL/cPVA microspheres demonstrates that the ASP oligomers endow the microspheres with bone binding properties. Figure 7C shows fluorescence microscopic images originating from pyrene loaded PCL/cPVA or PCL/cPVA-ASP on HAP surfaces. Increased intensity of the signal confirms a higher affinity between ASP functionalized microspheres and bone-like inorganic material, corroborating the data from Figure 7B.

### 3.6. Zone of Inhibition Established by PCL/cPVA-ASP

**Figure 8:**
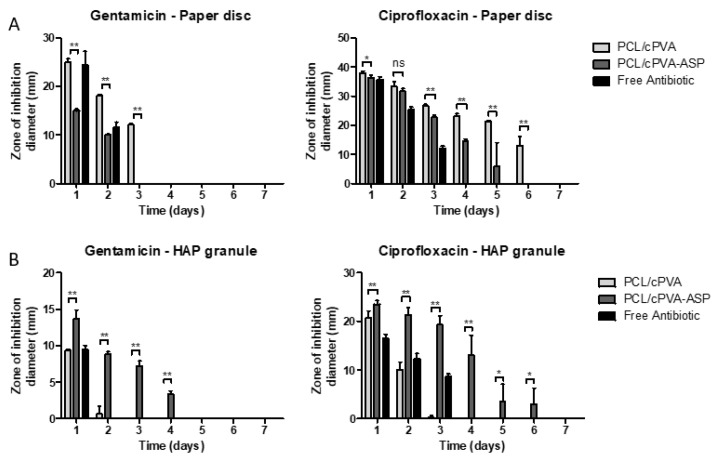
(**A**) Zone of inhibition established by 1 mg of PCL/cPVA or PCL/cPVA-ASP with antibiotic (GM-AOT, left and CF, right) load or the respective free antibiotic on paper discs (*n* = 4). (**B**) Zone of inhibition around a HAP granule exposed to GM-AOT or CF loaded microsphere dispersions or free antibiotic (*n* = 4). All GM-AOT loaded microspheres were tested against *S. aureus* while CF loaded microspheres were tested against *E. coli*. Error-bars represent standard deviations of quadruplicate measurements. Statistical test was a two-sample Student’s *t*-test (α = 0.01) with the following legend: * = *p* < 0.01, ** = *p* < 0.001, ns = *p* < 0.01).

The progression of the ZOI surrounding 1 mg of antibiotic-loaded PCL/cPVA or PCL/cPVA-ASP microspheres is showed in Figure 8A. From this figure, it can see that both PCL/cPVA and PCL/cPVA-ASP microspheres show a ZOI for both embedded antibiotics. However, it can be deduced that equal quantities of PCL/cPVA microspheres would establish a larger ZOI for a longer duration compared to PCL/cPVA-ASP that underwent further surface functionalization. This can be expected due to the loss of drug load during the functionalization procedure. The combined bone-binding and antimicrobial functions are presented in the form of inhibitory zone measurements of HAP granules that were exposed to suspensions of antibiotic loaded PCL/cPVA or PCL/cPVA-ASP microspheres. Figure 8B shows the ZOI established surrounding HAP granules after exposure to PCL/cPVA or PCL/cPVA-ASP. Due to the enhanced affinity of PCL/cPVA-ASP compared to PCL/cPVA, a larger and more persistent ZOI could be observed around the HAP granule for ASP functionalized microspheres with GM-AOT and CF. The microspheres without bone targeting moieties were able to establish an inhibition zone around the HAP granule on the first or second day due to non-specific adherence of small amounts of PCL/cPVA microspheres, matching with the findings observed during our HAP affinity assay (Figure 7). However, negligible zones of inhibition were observed for adhering PCL/cPVA microspheres at later timepoints. In contrast, when microspheres were functionalized with ASP, a larger ZOI diameter could be observed that was observable for up to 4 (GM-AOT) to 6 days (CF). The ZOI observations from Figure 8A,B and the fluorescent imaging from Figure 7C validate that microspheres with ASP surface functionalization exhibit enhanced affinity to HAP granules and release increased amounts of antibiotic in the vicinity of HAP granules.

## 4. Discussion

The relatively high recurrence rate of bone infection despite appropriate therapy suggests that improvements in antibiotic delivery to the infection site are required. Antibiotic carriers currently applied in clinics possess suboptimal properties such as non-biodegradability, incomplete antibiotic release, or excessive burst release of antibiotic load. The ZOI of antibiotic loaded PMMA bone cements and collagen sheets have been reported in our previous work, showing that PMMA cements release antibiotics over 7 days while collagen materials could only establish a ZOI for 3 days [18]. However, these frequently utilized antibiotic carriers come in bulky dimensions, necessitating invasive surgeries while preventing antibiotic release directly at the interface of the bone. In this work we introduced an antibiotic microsphere carrier system that could efficiently bind to calcified materials and release its antibiotic payload in close vicinity to infected bone mimics.

In our previous investigations, the bone targeting moiety was conjugated to COOH groups on the surface of the PCL microspheres, which were generated by alkaline hydrolysis (saponification) of PCL in 0.1M NaOH solutions. Due to hydrolytic degradation in presence of the such high pH levels, this procedure denatured the embedded GM-AOT and reduced its antimicrobial capacities. As many other antibiotics do not tolerate such extreme alkaline environments, our current motivation was to adjust the functionalization strategy by including functionalized cPVA surfactants in order to enrich the surface of the microspheres with COOH groups and omitting the harsh alkaline treatment procedure. While cPVA has been investigated for other applications (e.g., in relation with the modification of PVA crosslinking properties [31,32]), no reports have been found where cPVA was implemented as a surfactant for drug delivery purposes. Additionally, ASP oligomers were utilized as bone targeting moieties in this work opposed to ALN which showed impairment of osteoclast functionality in our previous work [18] and is associated with BRONJ pathology [24]. PCL/cPVA-ASP microspheres showed increased affinity to HAP granules compared to PCL/PVA microspheres and retained its antimicrobial properties after ASP functionalization. These results highlight the advantages of the use of cPVA surfactants instead of alkaline induced COOH formation for subsequent surface functionalization.

The investigation of drug release kinetics is essential when assessing novel drug delivery systems. For Korsmeyer–Peppas modeling of drug release, an *n*-value below 0.43 correlating to drug release by means of Fickian diffusion is expected for polydisperse microsphere samples, [33,34] as is indeed the case in this work. The main characteristics of Fickian diffusion are defined by a slow polymer relaxation and a high diffusion rate of solvent to the interior of the microsphere. Polymers with a glass transition temperature (T_g_) below the environmental temperature are often characterized by a Fickian diffusion of the drug from their polymeric matrix [30]. As the T_g_ of PCL is −60 °C and the microspheres were incubated at 37 °C, this statement conforms to our findings. Polymer crystallinity is another parameter that influences drug release kinetics from polymer materials. In oil/water emulsion systems, solvent evaporation rate has been known to influence the crystalline structure of the formed microspheres [35]. In this work, DCM was used as an organic oil phase, allowing for quick solvent evaporation and thus lower crystallization. With GM-AOT release over a 2-week period, this solvent choice and its effect on polymer crystallinity appears to be matching well with the sustained release kinetics desired for infection treatment.

Gentamicin salts (GM) and CF were used in this work as they are commonly applied in orthopedic surgery as a prophylactic agent or as a treatment for infections [36,37]. GM and CF are both active against Gram-positive and Gram-negative bacteria and can be considered broad-spectrum antibiotics. As GM is a hydrophilic antibiotic which is challenging to embed in polymer microspheres fabricated by O/W emulsions, we have previously applied a hydrophobic ion pairing method to substitute the sulphate ions of GM with a hydrophobic dioctyl sulfosuccinate counter-ion, resulting in the synthesis of water-insoluble GM-AOT [14,18]. This modified antibiotic could be efficiently embedded in hydrophobic matrices and showed similar antimicrobial properties compared to GM [18]. Additionally, the antibiotic CF was also interesting for investigation in drug delivery to bone as its molecular structure allows for chelation to Ca^2+^ [38] and can thus potentially interact with bone after being released from the microspheres. This interaction between CF and HAP was shown to be reversible at 37 °C and CF release from HAP composite materials was monitored in previous studies [39]. This characteristic of CF can potentially prolong the residence time of the antibiotic in bone tissue.

Even though both GM-AOT and CF loaded microspheres showed bactericidal properties (Figure 6), a clear difference in EE% and DL% of GM-AOT and CF was observed. Lower CF loading into the microspheres could be due to the lower solubility of CF in the DCM/acetic acid co-solvent system and different miscibility properties in the subsequent emulsification within the aqueous phase. To optimize CF encapsulation a W_1_/O/W_2_ emulsion could be considered, in which the CF load could be incorporated into the inner aqueous phase consisting of acidic solvents such as diluted acetic acid.

Comparative literature on drug delivery through peptide-based bone targeting is limited. Jiang et al. has performed a promising study on PLGA-PEG nanoparticles (NPs) functionalized with ASP and glycine (GLY) oligomers of six repeating units [27]. The oligomers were labeled with fluorescein isothiocyanate (FITC) in order to quantify the NPs binding affinity. Dispersions of ASP functionalized NPs were exposed to HAP containing hydrogels and NPs remaining in dispersion were quantified by light absorption measurements. Results showed that 85% (85 µg) of ASP functionalized NPs and 32% (32 µg) of GLY functionalized control NPs attached to the HA-hydrogel. This ratio between non-specific interaction of the GLY functionalized NPs and targeted interaction of the ASP functionalized NPs (1:2.65 ratio) is comparable to the results shown in Figure 7, where 103 µg of PCL/cPVA and 346 µg of PCL/cPVA-ASP (1:3.36 ratio) was quantified on HAP surfaces. As no information of the ASP configuration (d– or l–) was given by Jiang et al. it could be argued that the higher affinity ratio to calcified materials that we reported could be due to the non-hydrolysable D-ASP. The interaction between HAP granules and non-functionalized PCL/cPVA microspheres could originate from a combination of non-specific adsorption and from the carboxylated surface of the microspheres. Nap et al. investigated the complex formation between two carboxylate ions and ionic calcium [40], however, the complexation of COOH groups on the microspheres surface with Ca^2+^ ions in HAP crystals is not expected to significantly contribute to the interaction between the carboxyl-rich microspheres and HAP granules.

In a final experiment, the positive effect of the bone-binding surface was demonstrated via the larger and long-lasting inhibitory zone measurements of antibiotic loaded PCL/cPVA-ASP microspheres in comparison to PCL/cPVA microspheres loaded on HAP granules. Interestingly, even though CF encapsulation in PCL/cPVA-ASP microspheres (EE% = 12.76% ± 1.78%) was lower than the encapsulation of GM-AOT (EE% = 23.77% ± 1.39%), the ZOI that resulted from CF release was higher and persisted longer. This observation can be explained due to variability in sensitivity of *S. aureus* and *E. coli* bacterial strains against the two antibiotics. The minimal inhibitory concentration (MIC) of GM-AOT to the used *S. aureus* JAR strain was determined to be 0.98 µM [18], while the MIC of wild type *E. coli* is generally below 0.18 µM [41]. This difference in susceptibility could be the cause of the higher efficacy of CF loaded microspheres compared to those loaded with GM-AOT. Additionally, the molecular structure of CF allows for chelation through its carboxylic acid and ketone groups [42]. The release of chelated CF from a HAP/PCL nanocomposite substrate was previously reported and large quantities of CF (reported in absorbance values, but not correlated to a CF dosage) were detected in the release media over the course of 4 days at 37 °C [39]. Thus, it can be hypothesized that the CF intrinsic chelating ability to HAP combined with the sustained CF release from the PCL/cPVA-ASP microspheres bonded to the HAP, further enhance the local concentration of antibiotic at the bone-like material surface.

This work reports on a new approach towards carboxylation of microparticulate systems, implementing functionalized cPVA surfactants that facilitate further grafting of functional surface moieties. We believe that this strategy is of particular interest to a multitude of applications in targeted therapeutics. Specifically, the PCL/cPVA-ASP microspheres introduced in this work show to be a versatile drug delivery platform for antibiotic delivery to bone that, once further assessed in pre-clinical infection models, is open for clinical translation.

## 5. Conclusions

In this work we set out to develop a bone targeting antibiotic delivery system that was able to release antibiotic load in a sustained manner at the interface of calcified materials. The slow release of GM-AOT and CF antibiotics could reduce the optical density of bacterial suspensions and reduce bacterial titers by 5 log_10_ and 3.5 log_10_, respectively. PCL/cPVA-ASP microspheres showed a 3.36-times increased affinity to bone-mimicking surfaces compared to PCL/cPVA microspheres, ensuring that antibiotic release out of the microspheres occurs at the bone surface. Finally, we showed that PCL/cPVA-ASP microspheres could bind to HAP granules, surrogate for the inorganic bone matrix, and that a larger inhibition zone surrounding the granules could be observed for several days, in contrast to antibiotic loaded microspheres without ASP functionalization. In future studies, an assessment of the in vivo antimicrobial properties of the injectable bone targeting antibiotic loaded microspheres must be performed in a relevant animal OM model to confirm these in vitro findings.

## Figures and Tables

**Table 1:** Average size, polydispersity index (PDI), and antibiotic load characteristics of GM-AOT and CF loaded PCL/cPVA-ASP microspheres.

Loaded Antibiotic	Average Diameter ± Standard Deviation (SD) (µm)	Polydispersity Index (PDI)	Encapsulation Efficiency (EE%)	Drug Loading (%)
GM-AOT	6.03 ± 4.05 (*n* = 110)	0.450	23.77% ± 1.39%	4.75% ± 0.28%
CF	1.24 ± 0.76 (*n* = 103)	0.373	12.76% ± 1.78%	3.19% ± 1.53%

**Table 2:** Parameters of the linear fits from Higuchi and Korsmeyer–Peppas plots.

Released Antibiotic	Higuchi		Korsmeyer–Peppas	
	Higuchi Constant (*K_H_*)	Coefficient of Determination (R^2^)	*n*-Value (Slope)	Coefficient of Determination (R^2^)
GM-AOT	27.18	0.9923	0.38	0.9937
CF	22.72	0.9666	0.37	0.9948

## Supplementary Materials

The following are available online at https://www.mdpi.com/1999-4923/12/9/885/s1, Figure S1: OD_600_ measurements of bacterial suspensions and antibiotic loaded PCL/cPVA-ASP microsphere dispersions over 48 h.
