# Supplementary material for: Poly(Aspartic Acid) Functionalized Poly(ϵ-Caprolactone) Microspheres with Enhanced Hydroxyapatite Affinity as Bone Targeting Antibiotic Carriers
Source: Pharmaceutics. 2020 Sep 17;12(9):885. doi: 10.3390/pharmaceutics12090885 (PMC7559286; doi:10.3390/pharmaceutics12090885)
Supplement: Supplementary file 1 [file pharmaceutics-12-00885-s001.pdf]

pharmaceutics

Suplementary materials: Poly(Aspartic Acid) Functionalized Poly(ϵ-Caprolactone) Microspheres with Enhanced Hydroxyapatite Affinity as Bone Targeting Antibiotic Carriers

Stijn G. Rotman, Thomas F. Moriarty, Benjamin Nottelet, Dirk W. Grijpma, David Eglin and Olivier Guillaume

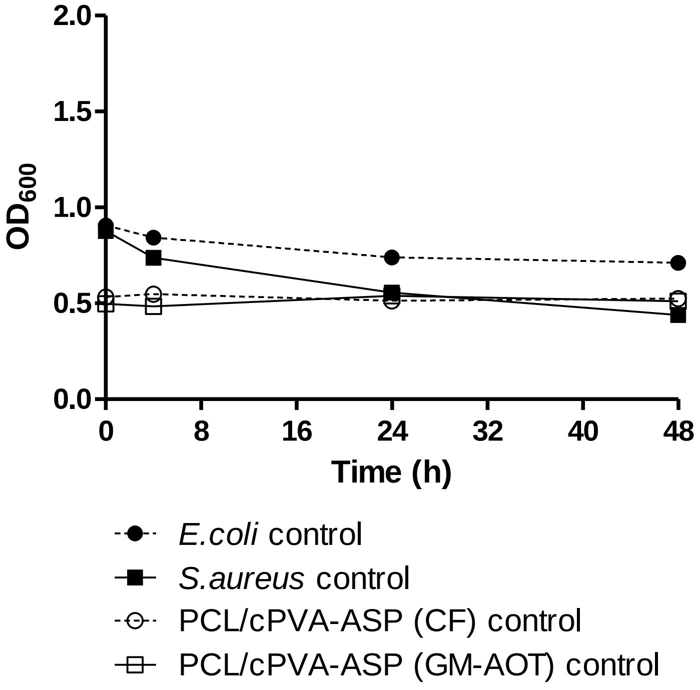

Figure S1. OD_{600} measurements of bacterial suspensions and antibiotic loaded PCL/cPVA-ASP microsphere dispersions over 48 hours.

OD600 of bacterial dispersions is slightly reduced over 48 hours in nutrient-poor PBS but is relatively stable for straightforward interpretation of antimicrobial effects of antibiotic loaded microsphere treatments. Dispersions of antibiotic loaded PCL/cPVA-ASP microspheres remain at a constant OD600 over the course of the experiment, simplifying interpretation of mixed dispersions of bacteria and microspheres in Figure 5.
